# Supplementary material for: Magnetic Nanocomposites of Coated Ferrites/MOF as Pesticide Adsorbents
Source: Molecules. 2022 Dec 21;28(1):39. doi: 10.3390/molecules28010039 (PMC9821965; doi:10.3390/molecules28010039)
Supplement: Supplementary file 1 [file molecules-28-00039-s001.zip › molecules-2100349-supplementary.pdf]

Supplementary Materials

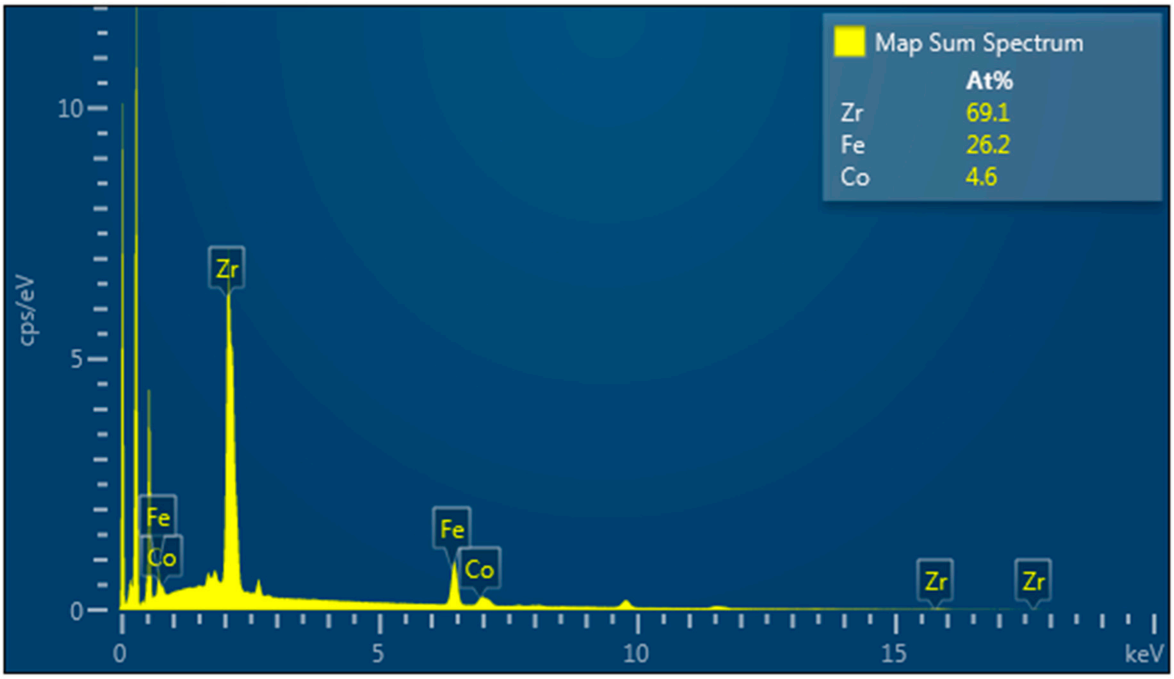

Figure S1. SEM/EDS spectrum of CF MMOF.

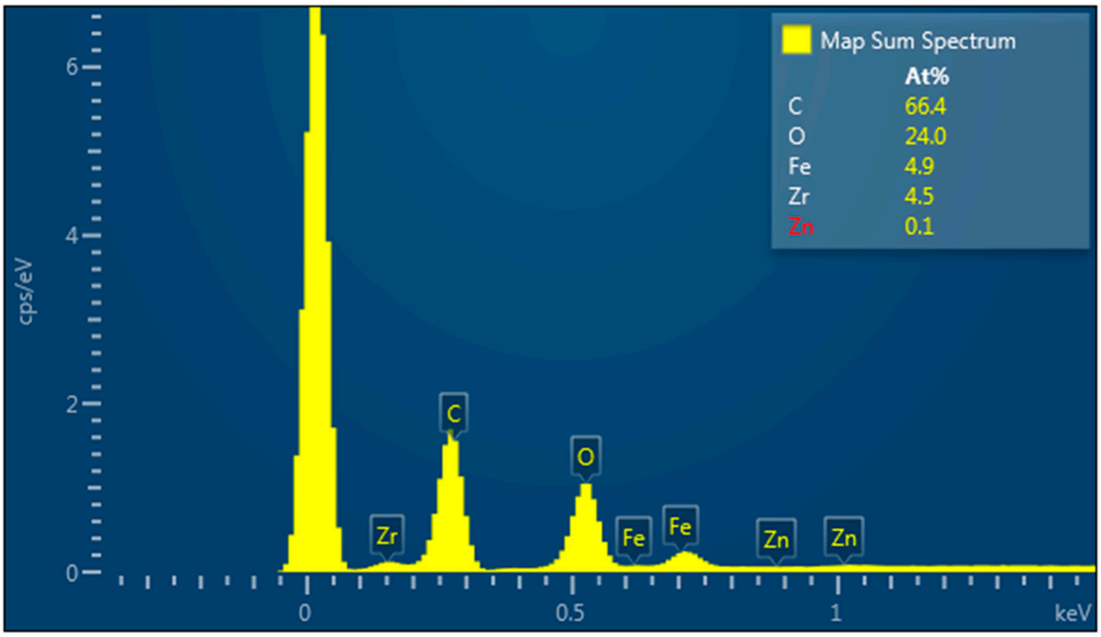

Figure S2. SEM/EDS spectrum of ZF(acac) MMOF.

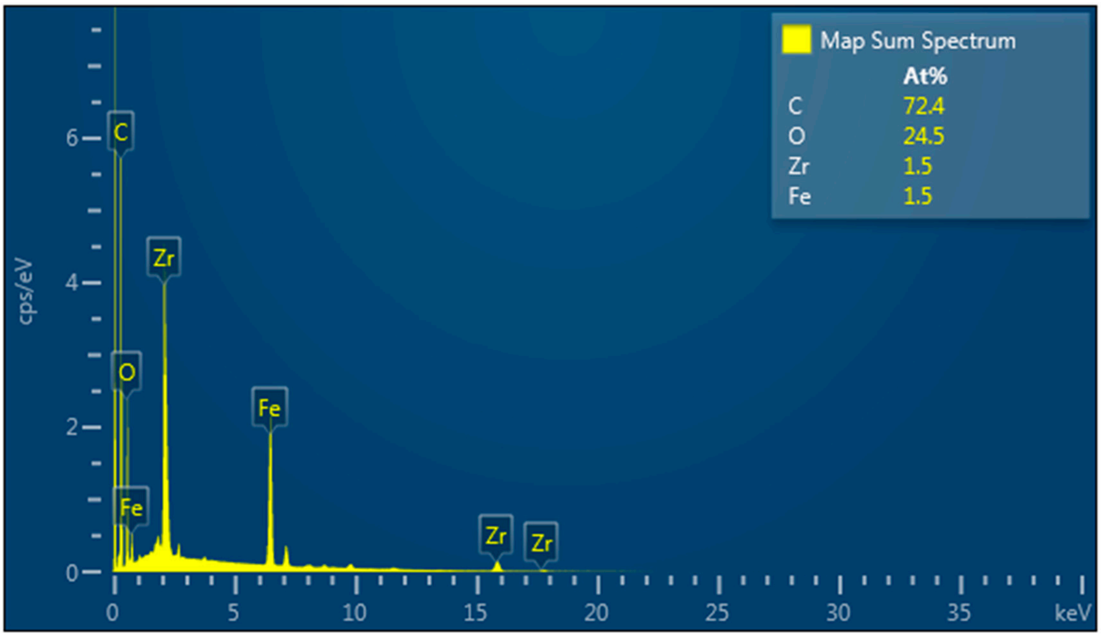

Figure S3. SEM/EDS spectrum of ZF(Cl) MMOF.

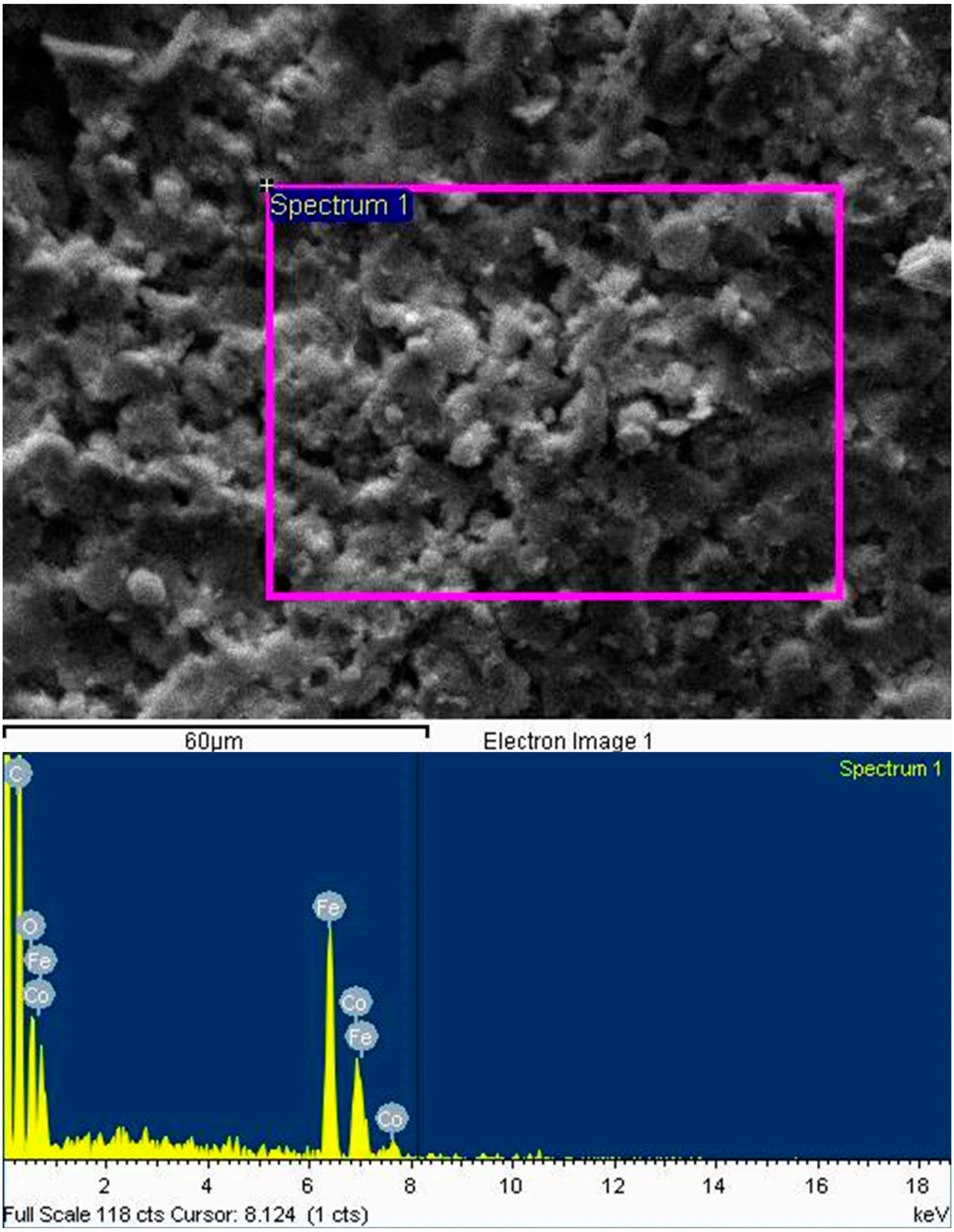

Figure S4. SEM/EDS of CF MNPs.

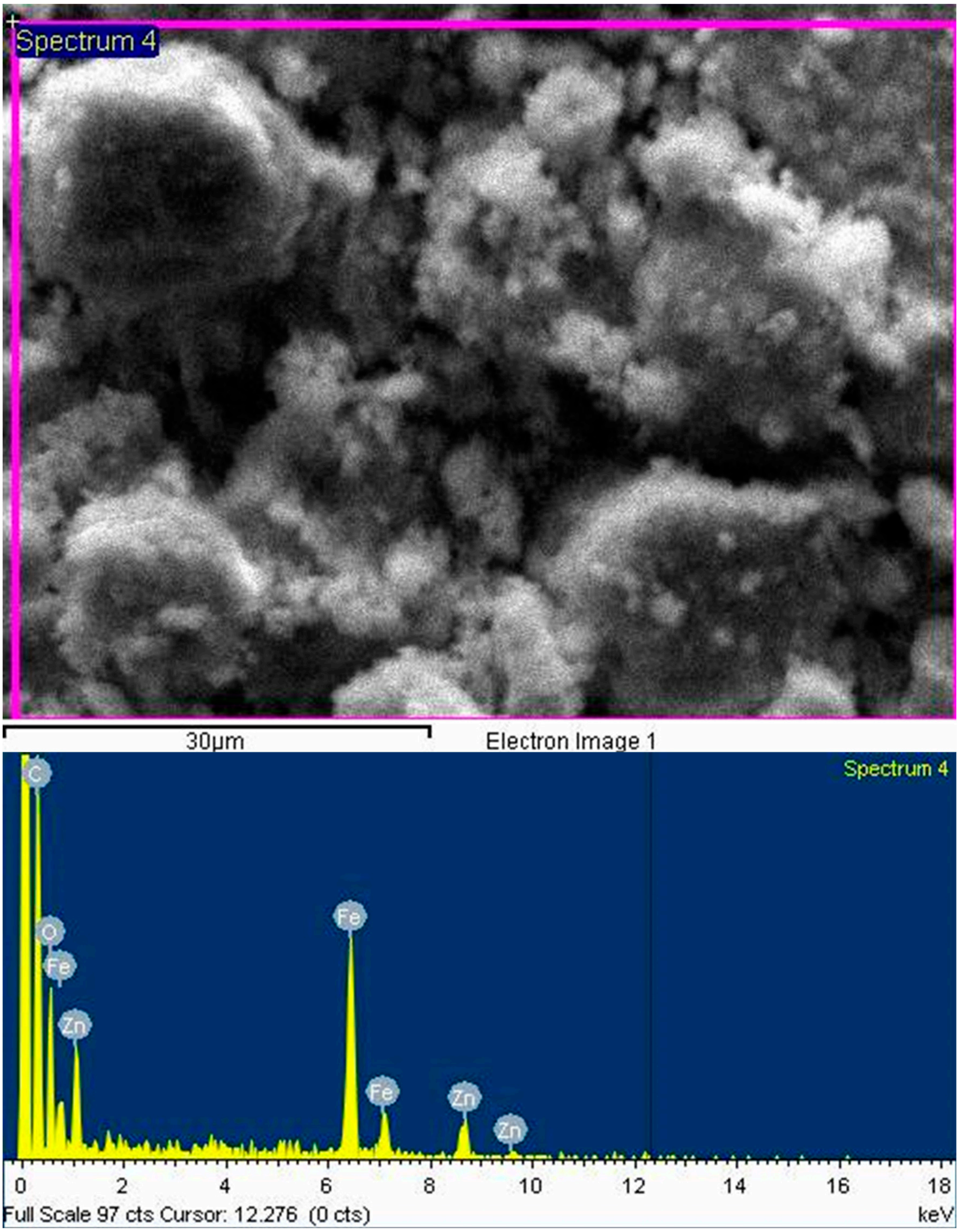

Figure S5. SEM/EDS of ZF(acac) MNPs.

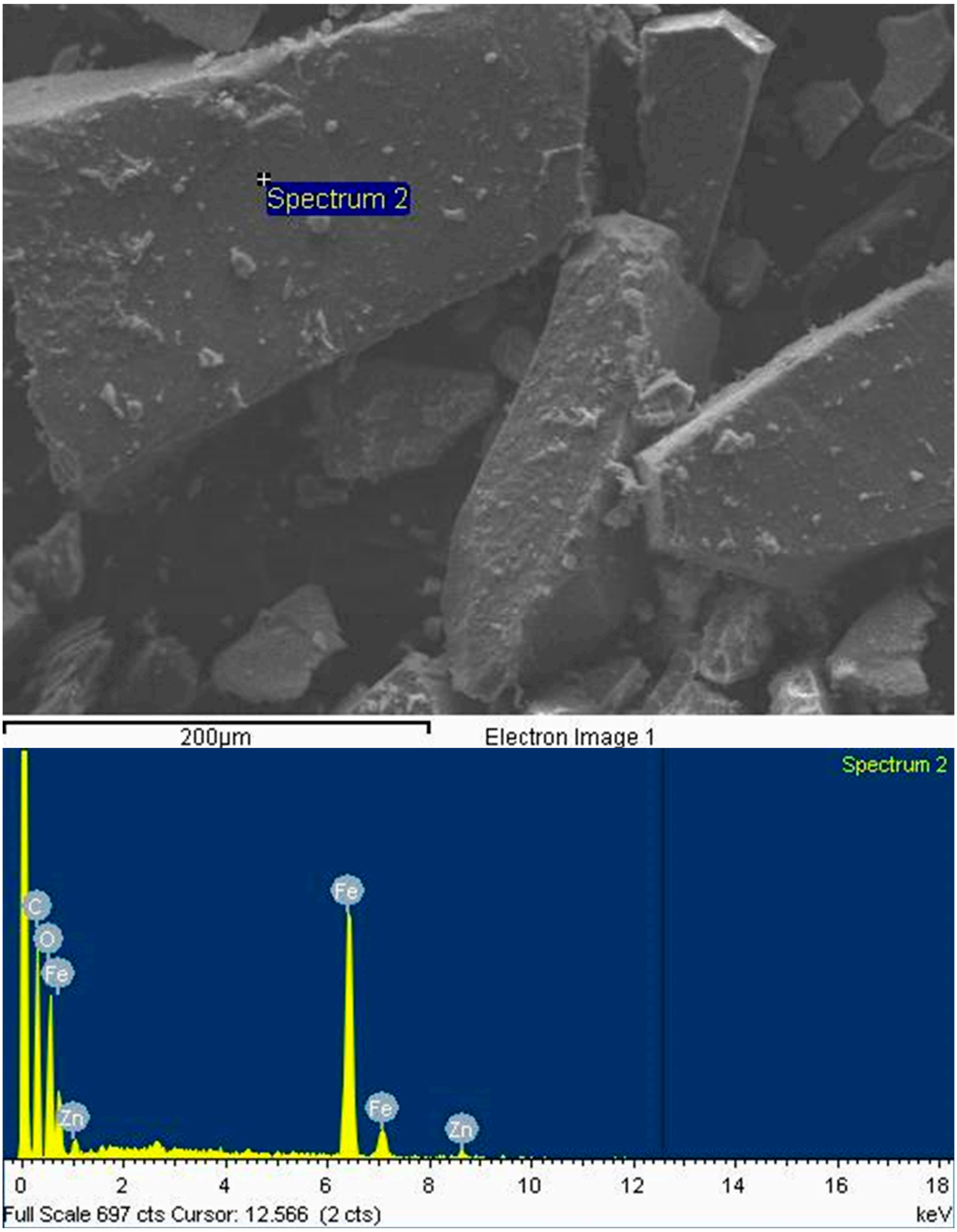

Figure S6. SEM/EDS of ZF(Cl) MNPs.

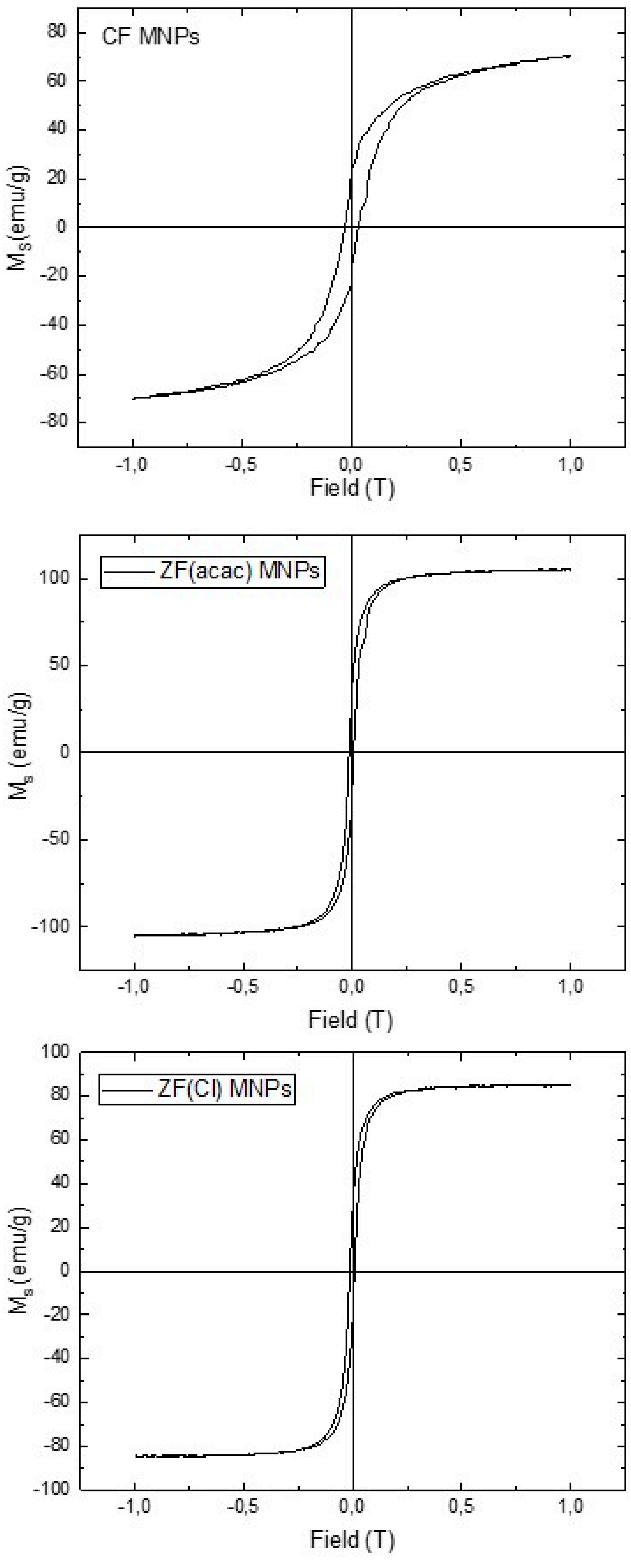

Figure S7.VSM hysteresis loops of CF, ZF(acac) and ZF(Cl) MNPs.

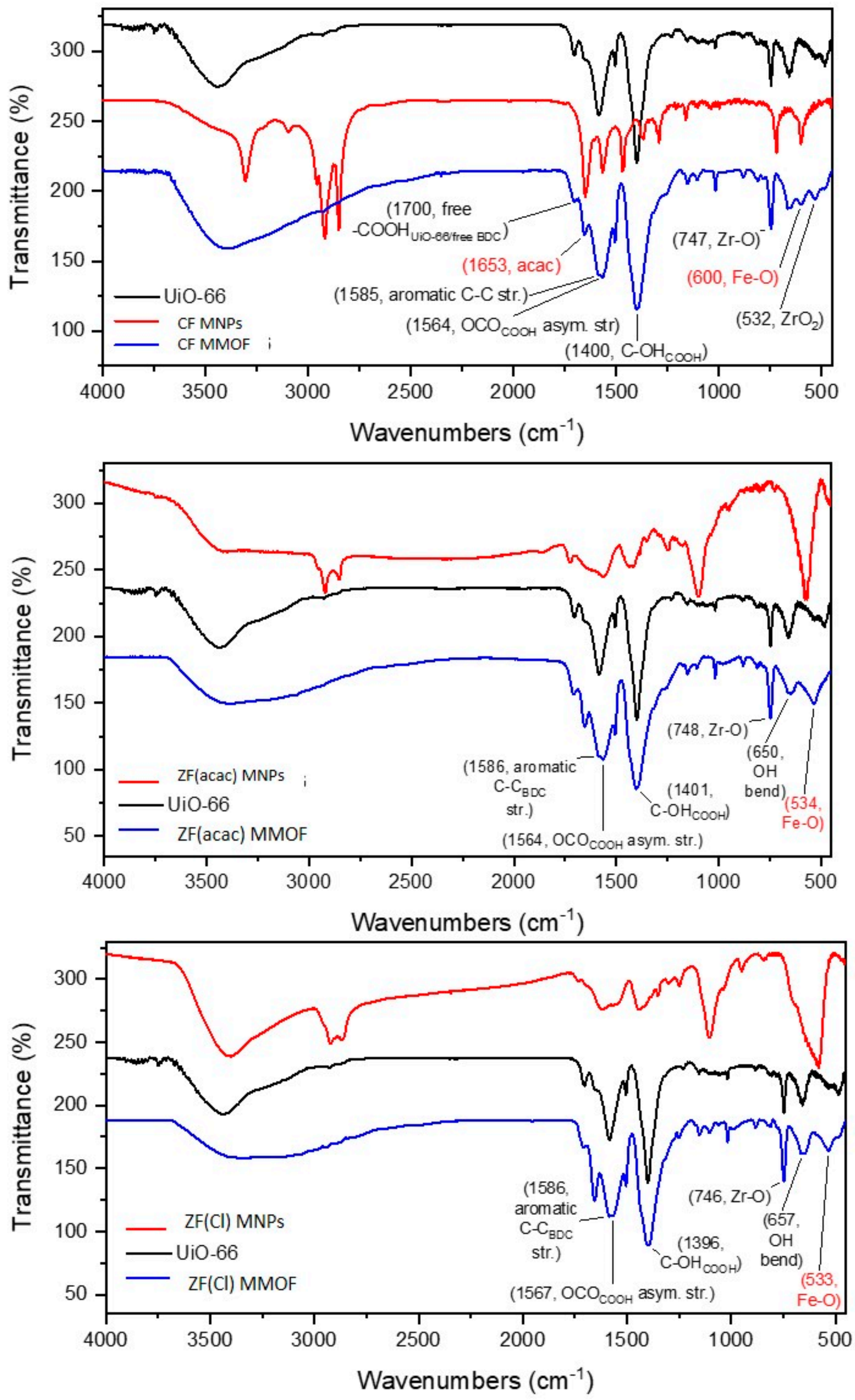

Figure S8. FTIR spectra for all MNPs and MMOFs.

Table S1. Recycle data after 24 hours of adsorption period for all three washed and dried MMOFs, in comparison with the 2,4-D starting solution curve.

	A(X1)	B(Y1)	C(X2)	D(Y2)	E(X3)	F(Y3)	G(X4)	H(Y4)
Long Name	Wavelengt	Absorbanc	Wavelengt	Absorbanc	Wavelengt	Absorbanc	Wavelengt	Absorbanc
Units	nm	a.u.	nm	a.u.	nm	a.u.	nm	a.u.
Comments		2,4-D stock		ZF(acac)@		ZF(Cl)@P		CF@ODA
F(x)=								
1	350	0,001	350	-0,002	350	-0,016	350	0,007
2	349	0,002	349	-0,001	349	-0,016	349	0,008
3	348	0,003	348	0	348	-0,017	348	0,008
4	347	0,002	347	-0,001	347	-0,018	347	0,008
5	346	0,003	346	-0,001	346	-0,018	346	0,009
6	345	0,002	345	0	345	-0,019	345	0,008
7	344	0,002	344	-0,001	344	-0,022	344	0,007
8	343	0,002	343	0	343	-0,022	343	0,007
9	342	0,003	342	0,001	342	-0,023	342	0,009
10	341	0,003	341	0,001	341	-0,024	341	0,008
11	340	0,003	340	0	340	-0,022	340	0,005
12	339	0,003	339	-0,001	339	-0,019	339	0,004
13	338	0,002	338	-0,002	338	-0,02	338	0,004
14	337	0,002	337	-0,002	337	-0,02	337	0,003
15	336	0,002	336	-0,001	336	-0,018	336	0,005
16	335	0,002	335	-0,002	335	-0,018	335	0,004
17	334	0,002	334	-0,001	334	-0,019	334	0,004
18	333	0,003	333	0	333	-0,018	333	0,005
19	332	0,002	332	-0,002	332	-0,018	332	0,004
20	331	0,003	331	-0,002	331	-0,018	331	0,005
21	330	0,003	330	0,001	330	-0,017	330	0,005
22	329	0,004	329	-0,001	329	-0,018	329	0,005
23	328	0,004	328	0	328	-0,017	328	0,005
24	327	0,004	327	0,001	327	-0,017	327	0,006
25	326	0,003	326	-0,001	326	-0,017	326	0,005
26	325	0,003	325	-0,001	325	-0,017	325	0,006
27	324	0,003	324	-0,001	324	-0,017	324	0,005
28	323	0,003	323	0,001	323	0,017	323	0,005
29	322	0,003	322	-0,001	322	-0,017	322	0,005
30	321	0,004	321	-0,001	321	-0,016	321	0,006
31	320	0,004	320	-0,001	320	-0,016	320	0,006
32	319	0,004	319	-0,001	319	-0,016	319	0,006
33	318	0,004	318	-0,001	318	-0,015	318	0,006
34	317	0,006	317	0	317	-0,015	317	0,008
35	316	0,004	316	-0,001	316	-0,015	316	0,007
36	315	0,006	315	0	315	-0,014	315	0,007
37	314	0,005	314	0	314	-0,015	314	0,007
38	313	0,006	313	0,001	313	0,014	313	0,008
39	312	0,007	312	0,002	312	-0,013	312	0,01
40	311	0,006	311	0,002	311	-0,013	311	0,01
41	310	0,007	310	0,003	310	-0,011	310	0,011
42	309	0,008	309	0,006	309	-0,009	309	0,013
43	308	0,009	308	0,009	308	-0,007	308	0,016
44	307	0,011	307	0,011	307	-0,005	307	0,019
45	306	0,014	306	0,016	306	-0,001	306	0,023
46	305	0,018	305	0,023	305	0,005	305	0,03
47	304	0,026	304	0,034	304	0,014	304	0,039
48	303	0,035	303	0,046	303	0,025	303	0,051
49	302	0,049	302	0,063	302	0,04	302	0,068
50	301	0,072	301	0,087	301	0,061	301	0,092
51	300	0,104	300	0,119	300	0,091	300	0,124
52	299	0,152	299	0,165	299	0,133	299	0,169
53	298	0,219	298	0,227	298	0,191	298	0,232
54	297	0,314	297	0,311	297	0,267	297	0,314
55	296	0,44	296	0,423	296	0,365	296	0,423
56	295	0,596	295	0,56	295	0,487	295	0,558
57	294	0,776	294	0,718	294	0,623	294	0,71
58	293	0,955	293	0,875	293	0,744	293	0,864
59	292	1,107	292	1,01	292	0,841	292	0,991
60	291	1,203	291	1,099	291	0,904	291	1,079
61	290	1,257	290	1,147	290	0,938	290	1,123
62	289	1,27	289	1,165	289	0,951	289	1,143
63	288	1,278	288	1,175	288	0,956	288	1,154
64	287	1,287	287	1,185	287	0,961	287	1,164
65	286	1,305	286	1,201	286	0,97	286	1,184
66	285	1,332	285	1,229	285	0,98	285	1,205
67	284	1,354	284	1,246	284	0,99	284	1,23
68	283	1,37	283	1,266	283	0,997	283	1,242
69	282	1,367	282	1,26	282	0,997	282	1,244
70	281	1,353	281	1,247	281	0,986	281	1,225
71	280	1,313	280	1,209	280	0,969	280	1,187
72	279	1,275	279	1,169	279	0,946	279	1,146
73	278	1,23	278	1,126	278	0,918	278	1,1
74	277	1,184	277	1,082	277	0,891	277	1,054
75	276	1,136	276	1,04	276	0,864	276	1,013
76	275	1,086	275	0,999	275	0,837	275	0,973
77	274	1,039	274	0,954	274	0,807	274	0,93
78	273	0,982	273	0,906	273	0,774	273	0,881
79	272	0,918	272	0,853	272	0,734	272	0,829
80	271	0,852	271	0,798	271	0,692	271	0,774
81	270	0,782	270	0,743	270	0,648	270	0,721
82	269	0,719	269	0,693	269	0,606	269	0,672
83	268	0,66	268	0,65	268	0,569	268	0,63
84	267	0,605	267	0,61	267	0,533	267	0,592
85	266	0,554	266	0,577	266	0,501	266	0,558
86	265	0,503	265	0,545	265	0,47	265	0,526
87	264	0,457	264	0,518	264	0,443	264	0,499
88	263	0,413	263	0,496	263	0,419	263	0,476
89	262	0,374	262	0,479	262	0,399	262	0,458
90	261	0,338	261	0,47	261	0,384	261	0,447
91	260	0,305	260	0,466	260	0,374	260	0,442
92	259	0,276	259	0,471	259	0,37	259	0,444
93	258	0,249	258	0,483	258	0,372	258	0,452
94	257	0,225	257	0,501	257	0,377	257	0,465
95	256	0,204	256	0,527	256	0,389	256	0,487
96	255	0,188	255	0,558	255	0,406	255	0,513
97	254	0,173	254	0,593	254	0,426	254	0,542
98	253	0,162	253	0,63	253	0,447	253	0,574
99	252	0,156	252	0,666	252	0,47	252	0,605
100	251	0,156	251	0,703	251	0,493	251	0,636
101	250	0,161	250	0,739	250	0,519	250	0,668
102	249	0,176	249	0,779	249	0,548	249	0,706
103	248	0,202	248	0,825	248	0,584	248	0,748
104	247	0,243	247	0,88	247	0,629	247	0,801
105	246	0,304	246	0,947	246	0,686	246	0,867
106	245	0,391	245	1,033	245	0,76	245	0,95
107	244	0,512	244	1,139	244	0,851	244	1,054
108	243	0,674	243	1,265	243	0,958	243	1,178
109	242	0,885	242	1,413	242	1,081	242	1,327
110	241	1,143	241	1,569	241	1,201	241	1,488
111	240	1,431	240	1,708	240	1,301	240	1,63
112	239	1,673	239	1,796	239	1,364	239	1,726
113	238	1,832	238	1,854	238	1,391	238	1,786
114	237	1,887	237	1,863	237	1,4	237	1,8
115	236	1,883	236	1,863	236	1,397	236	1,801
116	235	1,871	235	1,86	235	1,389	235	1,795
117	234	1,875	234	1,851	234	1,387	234	1,788
118	233	1,862	233	1,844	233	1,377	233	1,78
119	232	1,847	232	1,844	232	1,372	232	1,772
120	231	1,816	231	1,822	231	1,358	231	1,762
121	230	1,826	230	1,813	230	1,352	230	1,752
122	229	1,814	229	1,801	229	1,34	229	1,74
123	228	1,811	228	1,793	228	1,323	228	1,729
124	227	1,795	227	1,782	227	1,315	227	1,721
125	226	1,793	226	1,778	226	1,308	226	1,711
126	225	1,772	225	1,775	225	1,303	225	1,702
127	224	1,756	224	1,751	224	1,286	224	1,691
128	223	1,753	223	1,745	223	1,276	223	1,68
129	222	1,742	222	1,732	222	1,27	222	1,672
130	221	1,741	221	1,723	221	1,256	221	1,662
131	220	1,724	220	1,711	220	1,248	220	1,652
132	219	1,719	219	1,699	219	1,241	219	1,636
133	218	1,696	218	1,688	218	1,224	218	1,627
134	217	1,686	217	1,678	217	1,212	217	1,613
135	216	1,652	216	1,663	216	1,2	216	1,6
136	215	1,654	215	1,649	215	1,186	215	1,581
137	214	1,65	214	1,626	214	1,168	214	1,563
138	213	1,616	213	1,607	213	1,147	213	1,546
139	212	1,61	212	1,583	212	1,12	212	1,522
140	211	1,583	211	1,563	211	1,103	211	1,501
141	210	1,548	210	1,538	210	1,073	210	1,475
142	209	1,521	209	1,509	209	1,049	209	1,448
143	208	1,49	208	1,476	208	1,016	208	1,417
144	207	1,473	207	1,445	207	0,985	207	1,385
145	206	1,415	206	1,411	206	0,955	206	1,352
146	205	1,391	205	1,372	205	0,916	205	1,305
147	204	1,336	204	1,331	204	0,873	204	1,271
148	203	1,306	203	1,286	203	0,83	203	1,226
149	202	1,254	202	1,241	202	0,787	202	1,178
150	201	1,207	201	1,193	201	0,743	201	1,13
151	200	1,141	200	1,146	200	0,697	200	1,084
152								
153								
154								
155								
156								
